# TFinDit: transcription factor-DNA interaction data depository

**DOI:** 10.1186/1471-2105-13-220

**Published:** 2012-09-03

**Authors:** Daniel Turner, RyangGuk Kim, Jun-tao Guo

**Affiliations:** 1 Department of Bioinformatics and Genomics, College of Computing and Informatics, University of North Carolina at Charlotte, Charlotte, NC 28223, USA

**Keywords:** Transcription factor, Database, Binding site prediction, Interaction potential

## Abstract

**Background:**

One of the crucial steps in regulation of gene expression is the binding of transcription factor(s) to specific DNA sequences. Knowledge of the binding affinity and specificity at a structural level between transcription factors and their target sites has important implications in our understanding of the mechanism of gene regulation. Due to their unique functions and binding specificity, there is a need for a transcription factor-specific, structure-based database and corresponding web service to facilitate structural bioinformatics studies of transcription factor-DNA interactions, such as development of knowledge-based interaction potential, transcription factor-DNA docking, binding induced conformational changes, and the thermodynamics of protein-DNA interactions.

**Description:**

TFinDit is a relational database and a web search tool for studying transcription factor-DNA interactions. The database contains annotated transcription factor-DNA complex structures and related data, such as unbound protein structures, thermodynamic data, and binding sequences for the corresponding transcription factors in the complex structures. TFinDit also provides a user-friendly interface and allows users to either query individual entries or generate datasets through culling the database based on one or more search criteria.

**Conclusions:**

TFinDit is a specialized structural database with annotated transcription factor-DNA complex structures and other preprocessed data. We believe that this database/web service can facilitate the development and testing of TF-DNA interaction potentials and TF-DNA docking algorithms, and the study of protein-DNA recognition mechanisms.

## Background

Transcription factors (TFs) represent a distinct group of DNA binding proteins. They are sequence-specific while allowing certain degrees of variations at particular sites [1]. Though regulation of gene expression is a complicated biological process, one key step of this process is the binding of TFs to their DNA binding sites. At the genome level, identification of DNA target sites of transcription factors has been considered one of the grand challenges in post-genomic bioinformatics. The complex structures in Protein Data Bank (PDB) provide fine details about macromolecular interactions [2]. Knowledge of TF-DNA interactions can help us better understand the mechanisms of protein-DNA recognition, and more importantly, guide the design of new therapeutics for diseases in which transcription factors play critical roles [3-5]. Even though the number of TF-DNA complex structures in PDB has increased steadily due to technical advance in solving complex structures, it still only represents a small percentage of all the annotated transcription factors and their target DNA sites. At the same time, computational studies have made notable progress in modeling protein-DNA interactions. These include development of knowledge-based protein-DNA interaction potentials [6-8], investigation of binding affinity and specificity [9,10], and protein-DNA docking studies [11-13]. Recently, structure-based TF binding site prediction has received much deserved attention owing to its ability to consider the position interdependence of TFs and the contribution of flanking sequences to binding specificity. The development of more accurate interaction potentials makes these structure-based methods feasible and more appealing in computational prediction of TF binding sites [8,11,14].

The paramount importance of transcription factors in gene regulation has attracted significant interests and efforts in developing TF resources either for one specific genome, such as RegulonDB for *E. coli* K-12 [15] and EDGEdb for *C. elegans*[16], or for one specific kingdom, such as JAPAR for Eukaryotes [17] and RegTransBase for bacteria [18]. The TF resources currently available across the tree of life are listed in a recent survey [19]. Most of these TF resources have either manually annotated or computationally predicted TFs while others use a combination of both annotation approaches. Though these TF resources contain large amounts of data that are valuable to study the diversity and evolution of transcription factors, they are not designed for structural bioinformatics studies of TF-DNA interactions.

On the other hand, several databases/web servers about general protein-nucleic acids interactions have been developed. These include AANT [20], ProNIT [21], NPIDB [22], PDA [23], BIPA [24], hPDI [25], 3D-footprint [26], PDIdb [27], ccPDB [28] and others. While each database/web server offers search options on certain aspects about general protein-nucleic acid interactions, the unique characteristics of transcription factors and the imperative goal of structure-based TF-binding site prediction call for a TF-specific database/web server, especially when transcription factors are not well classified and annotated in PDB. In addition, previous studies have revealed different interaction “modes” between transcription factors and other types of DNA binding proteins [29,30]. To the best of our knowledge, there are no TF-specific structural databases/web services available.

We developed TFinDit (for Transcription Factor-DNA interaction Data depository) to facilitate structural bioinformatics studies of TF-DNA interactions. TFinDit offers annotated TF-DNA complex structures and other useful information, such as unbound TF structures, thermodynamic data of TF-DNA complexes, and automatic mapping between TF-DNA complexes and known TF binding sites. TFinDit also provides a web interface with multiple search options. Potential users can generate datasets based on their research needs in studying TF-DNA interaction, such as bound-unbound TF pairs, DNA binding sites, and thermodynamic data for wild-type and/or mutants (TF and DNA), or focus on the structural details of one specific TF-DNA complex. The framework of TFinDit can be easily extended to include more useful information once identified in the future.

## Construction and content

Computationally, TFinDit has two major components: a relational database using MySQL 5.0.45 and a web server providing an interface accessible to potential users to search the database and display the search results. The web server is developed with a combination of PHP 5.1.6, Java JDK v1.6.0, Python 2.4.3, and Apache Web Server 2.3.3.

The database contains all TF-DNA complexes from PDB [2]. The collection of TF-DNA complexes from PDB is not trivial since the classification of some DNA-binding proteins in PDB is ambiguous. For example, transcription factors *Escherichia coli* SigmaE Region 4, 2H27 [31] and the ribbon-helix-helix domain of *Escherichia coli* PutA, 2RBF [32] are classified as “transferase” and “oxidoreductase” respectively in PDB. So we first developed an in-house program that can automatically identify transcription factors in PDB by combining information from Gene Ontology (GO) terms [33], PDB keywords, and UniProt keywords [34]. The procedure of the annotation process is shown in Additional file 1 Figure S1. The script and related files are available for download from the TFinDit site (Resources Tab).

**Figure 1:**
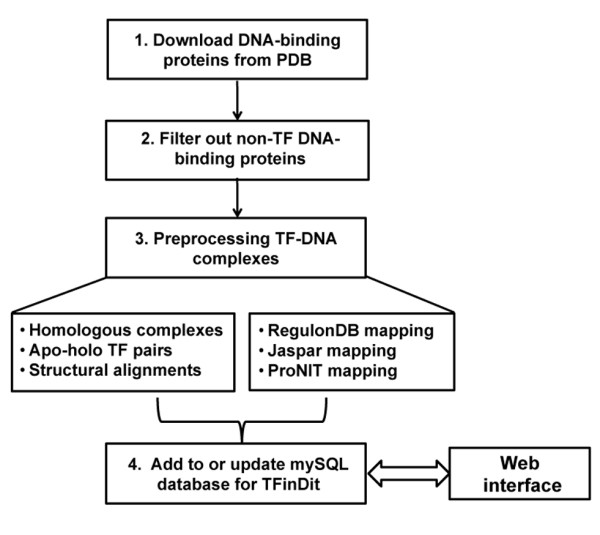
Procedure for TFinDit construction and update.

The procedure for generating the initial data and for future updates is shown in Figure 1. Briefly, all the DNA-binding proteins are culled from PDB. The TF-DNA complexes with double-strand DNA are selected using our in-house TF-annotation program that takes PDB IDs as inputs. The list of TF-DNA complexes will serve as the base for getting other data and for preprocessing. The first step in preprocessing is to search for homologous TF-DNA complexes and homologous TF structures in free state (unbound structures) with at least 80% sequence identity to the query bound TF structures. Data from both the sequential (similarity, coverage, etc.) and structural comparisons are stored in the database (Figure 1). TF structural comparison is carried out with TM-align that uses TM-score for alignment optimization [35-37]. The TM-score is normalized independent on the protein's size and is more sensitive to global structure changes than to local structure changes compared to RMSD (Root Mean Square Deviation) [35]. While RMSD is a widely used metric for structural differences, TM-score is more suitable for spotting global structure changes [35-37]. In addition, previous studies have shown that the activation regions of transcription factors have higher degree of disorder and transcription factors in eukaryote have more disordered regions than those in prokaryote [38-40]. Neither TM-score nor RMSD could reflect the structural differences caused by missing residues or disordered regions in TF structures. After structural alignment, both the TM-scores and the RMSD values are calculated using the C-alpha of the amino acids between the unbound and bound TF structures and are stored in the database. Currently, the database contains 1391 bound and 2370 unbound chains.

Another important component in preprocessing is the mapping of TF structures to entries in other important databases. These include databases with TF binding sites (RegulonDB and Jaspar) [15,17] and ProNIT, a thermodynamic database for protein-nucleic interactions [21]. Among the 1391 bound TF chains in current release, 307 have ProNIT entries and 433 have annotated binding sequences from RegulonDB/Jaspar. After the preprocessing step, all the data are stored in a relational database. The same procedure will be used for future updates and newly identified entries and related data will be added to the database (Figure 1). We plan to update the database every two to three months.

## Utility and discussion

**Figure 2:**
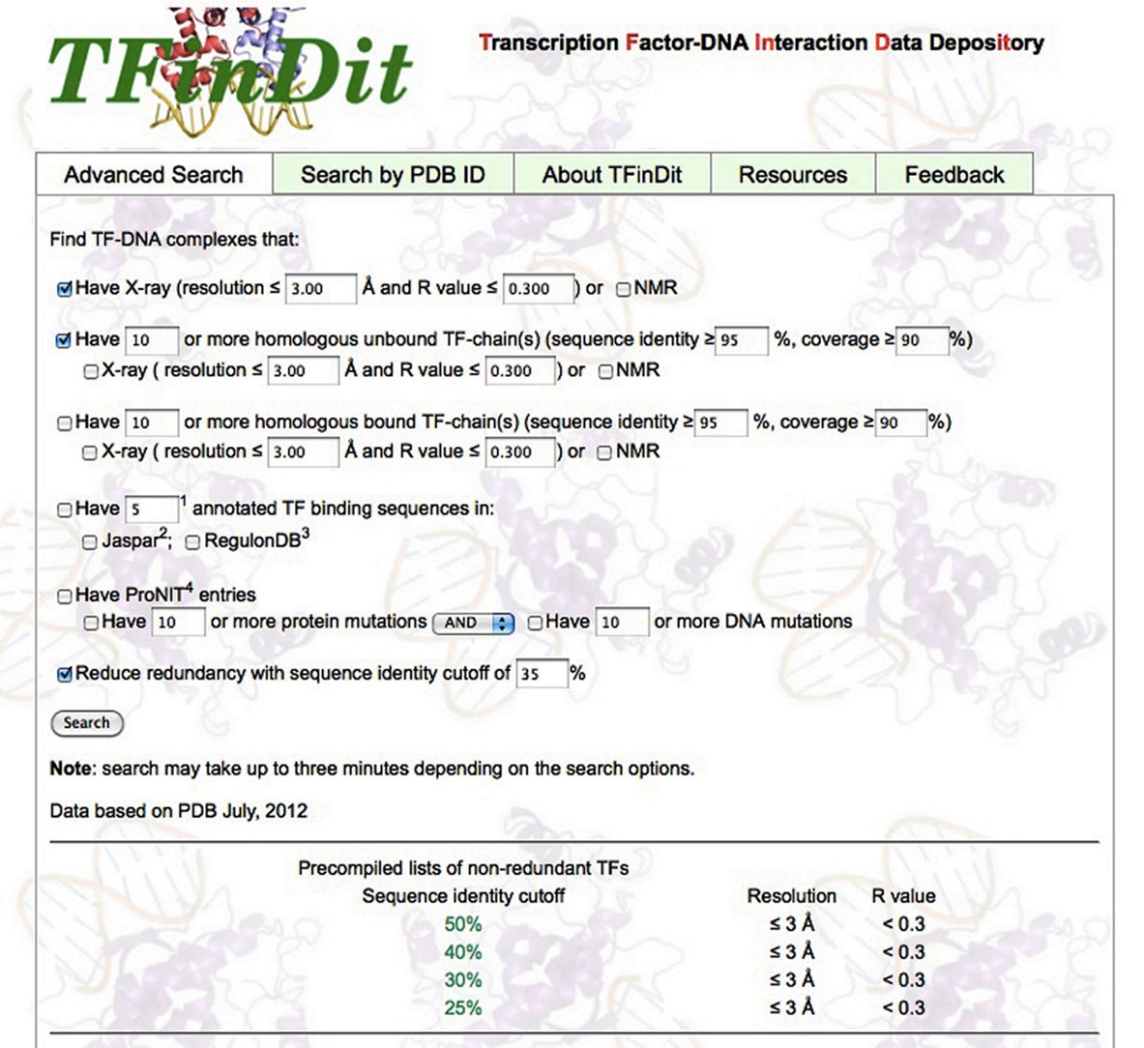
A snapshot of the “Advanced Search” page for TFinDit.

The web interface offers two options for queries. One is for culling non-redundant datasets for different research purposes. For example, users can generate a non-redundant dataset of bound-unbound pairs for studying conformational changes after TF-DNA binding or docking studies. Other useful datasets that can be generated include homologous TF-DNA complexes, TF-DNA complexes with thermodynamic data for both wild-type and/or mutant molecules, and TF-DNA complexes with experimentally validated binding sequences (Figure 2). Users can specify the resolution for x-ray structures, the sequence identity and coverage for homologous sequences, and the minimum number of entries that satisfy the selection criteria. PISCES is used to remove redundancy [41].

**Figure 3:**
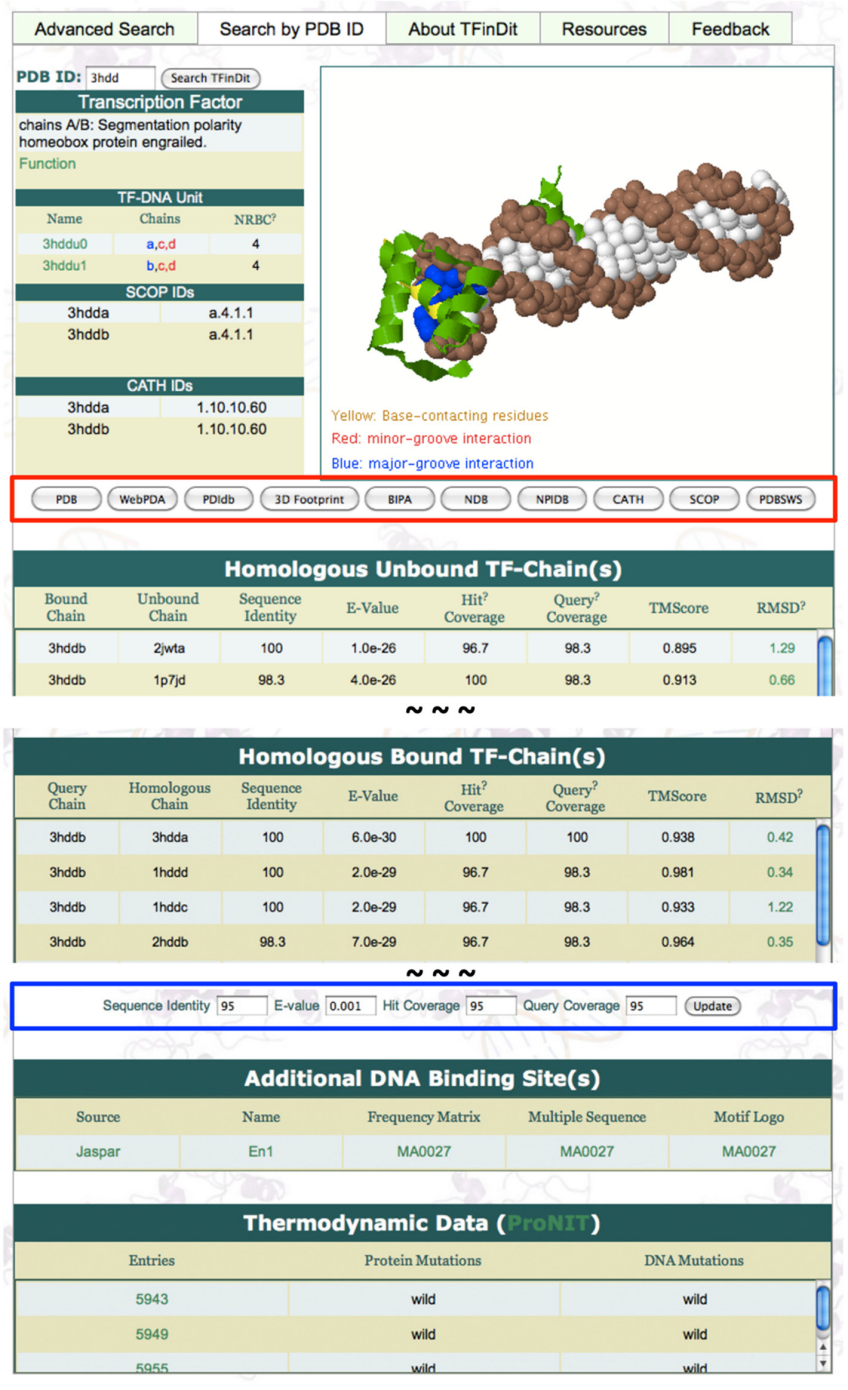
**Detailed information for TFinDit entry 3HDD. **The red box indicates the quick links to other analysis tools. The blue box shows the cutoff values that users can change and get updated data.

The other search option allows the retrieval of detailed structural and related data for a specific TF-DNA complex in TFinDit. An example for PDB ID 3HDD [42] is shown in Figure 3. These data include the homologous unbound transcription factors, homologous TF-DNA complexes, known annotated additional binding sequences, and thermodynamic data for the wild-type and mutants of the complexes in ProNIT (Figure 3). The sequence identity, coverage, and the structural differences between homologous bound-unbound or bound-bound pairs in terms of both the TM-Score measure and RMSD, are also displayed. Users also have the option to change the cutoffs for sequence identity, *E*-value, coverage (Blue Box in Figure 3). In addition, links of the TFinDit entry to other useful web services are also provided (Red Box in Figure 3). These include PDB [2], WebPDA [23], PDIdb [27], 3D-footprint [26], BIPA [24], NDB [43], and NPIDB [22] and to structural classifications websites CATH [44] and SCOP [45]. Users can get a quick access to all the related predictive or analysis tools for each TF-DNA entry from TFinDit. On the “Resources” page, a number of useful predictive tools for modeling TF-DNA interactions and other services are provided and the list will be updated when more tools are identified. Current tools include *TF-Modeller* for building comparative TF-DNA complex models [46] and DDNA3 for DNA binding domain prediction [47], our in-house program for TF annotation, and some services listed in the quick-link box (Figure 3).

## Conclusions

TFinDit is a specialized structural database with annotated transcription factor-DNA complex structures and other related data. We believe that this database/web service can facilitate structural bioinformatics studies, especially in the development of TF-DNA interaction potentials, the testing of TF-DNA docking algorithms, and the study of protein-DNA recognition mechanisms.

## Availability and requirements

The service is available at http://bioinfozen.uncc.edu/tfindit

## Abbreviations

PDB: Protein Data Bank; RMSD: Root Mean Square Deviation; TF: Transcription Factor.

## Competing interests

The authors declare that they have no competing interests.

## Authors’ contributions

DT implemented the database and the web service. RK participated in the design and the initial implementation of TFinDit. JTG conceived the study, participated in the design, and wrote the manuscript. All authors read and approved the final manuscript.

## Supplementary Material

Additional file 1 Figure S1. Flowchart for identifying TF-DNA complexes in PDB. Click here for file
