# Supplementary figures and images for: TFinDit: transcription factor-DNA interaction data depository
Source: BMC Bioinformatics. 2012 Sep 3;13:220. doi: 10.1186/1471-2105-13-220 (PMC3483241; doi:10.1186/1471-2105-13-220)

Figure S1. Flowchart for identifying TF-DNA complexes in PDB.

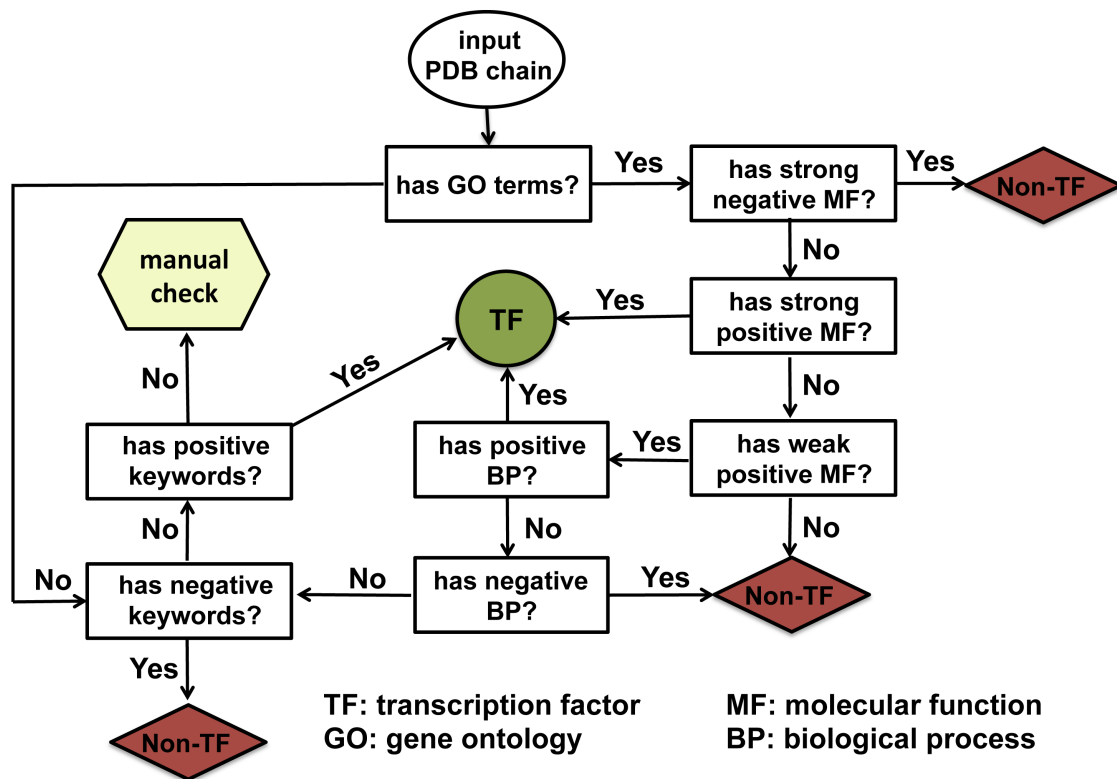

Supplement: Additional file 1 — Figure S1. Flowchart for identifying TF-DNA complexes in PDB. [file 1471-2105-13-220-S1.pdf]
